# Comprehensive Analysis of Risk Factors and Prognostic Outcomes in Cases of Cerebral Venous and Sinus Thrombosis in a Tertiary Care Setting

**DOI:** 10.7759/cureus.68574

**Published:** 2024-09-03

**Authors:** Awais Ilyas Mohammed, Nishanth Gejjalagere Chandrashekar, Reemu Bansal, Naresh Vishwanath Iyer Murali, Tejaswee Lohakare

**Affiliations:** 1 General Medicine, United Lincolnshire Hospital Trust, Grantham, GBR; 2 Emergency Medicine, United Lincolnshire Hospital Trust, Grantham, GBR; 3 Neurosurgery, Marengo Asia Hospital, Faridabad, IND; 4 Child Health Nursing, Smt. Radhikabai Meghe Memorial College of Nursing, Wardha, IND

**Keywords:** cerebral venous thrombosis (cvt), cerebral venous and sinus thrombosis, contributing factors, mrs rating, increased red blood cell count, cerebrovascular accident, cerebral vein and sinus clotting

## Abstract

Background

Cerebral venous and sinus thrombosis (CVT) is one of the most common causes of stroke in young people. With timely diagnosis and the right medical attention, this relatively rare neurologic condition may be curable. Finding the risk variables and outcome determinants is the aim of this study.

Methodology

A two-year prospective observational research was carried out in a tertiary care facility. Notable were the patient's demographics, symptomatology, and risk factor history. The Modified Rankin Scale (mRS) was employed to assess the patient's outcome and prognosis both at admission and after six weeks. The mRS scores at admission and follow-up were compared concerning outcome factors using the chi-square test.

Results

In all, there were 75 people with CVT. More men (42 patients, 56%) than women (33 patients, 44%), particularly in their third decade, were impacted. Polycythemia (22 patients, 29.3%) was the most prevalent risk factor, followed by the use of oral contraceptives (14 patients, 18.7%). Based on their mRS scores upon entry, 38 individuals (50.7%) were classified as functionally independent (mRS < 2), whereas 37 individuals (49.3%) were deemed functionally dependent (mRS > 2). At the six-week follow-up, 54 patients (72%) were functionally independent. Decompressive craniotomies were performed on 15 patients (20%), of which 10 (13.33%) had improvement, two (2.67%) had deterioration, and one patient passed away. The percentage of deaths was 1.33%. Two patients (2.67%) were not followed up with.

Conclusion

The present findings highlight that CVT predominantly affects younger individuals with a slight male predominance. The leading risk factors were polycythemia and the use of oral contraceptive pills (OCPs). Despite generally favorable prognoses with appropriate management, poorer outcomes were linked to altered consciousness, neurological deficits, and intracerebral hemorrhage (ICH) at presentation.

## Introduction

Cerebral venous and sinus thrombosis (CVT) is a vascular disorder characterized by the formation of thrombi in the brain's venous pathways, such as the dural venous sinuses and the veins of the brain's cortex. Unlike more common forms of stroke caused by arterial blockages, CVT involves the venous system, which can lead to a range of neurological symptoms due to increased intracranial pressure, venous infarction, and hemorrhage [1]. Cerebral venous and sinus thrombosis accounts for approximately 0.5% of all stroke cases globally, highlighting its rarity in the broader context of cerebrovascular diseases. The annual incidence of CVT is estimated to be between three and four cases per one million population [2]. Despite its relatively low incidence, CVT is a significant health concern due to its potential impact on younger individuals [1, 2].

In India, strokes in young adults constitute almost 30% of all stroke cases, highlighting the significant impact of cerebrovascular diseases on this age group. Within this subset, CVT represents 10% to 20% of cases, indicating that it is a considerable contributor to stroke in the young population in India [3]. The presentation of CVT can vary widely, from mild headaches to severe neurological deficits, making its diagnosis and management a complex challenge for healthcare providers [3]. One of the distinctive features of CVT compared to arterial strokes is its relatively lower mortality rate. This is promising because most CVT patients have favorable long-term outcomes with proper treatment [4]. Progress in neuroimaging and therapeutic techniques has greatly enhanced the prognosis for CVT patients, resulting in improved management and fewer complications [4,5]. Despite the advances in understanding and treating CVT, there is still limited research focusing specifically on the Indian population. There is a scarcity of comprehensive studies analyzing the contributing factors, results, and predictive indicators related to cerebral venous thrombosis in India. This gap in research is significant, as the Indian population may have unique risk profiles and outcomes that differ from those observed in other regions [6].

This study aims to fill this gap by evaluating CVT's risk factors and outcome determinants in Indian patients. By analyzing a cohort of individuals diagnosed with CVT, the research seeks to identify prevalent risk factors, assess both short-term and long-term outcomes, and determine prognostic indicators to inform clinical practice. This research is crucial for developing tailored interventions and improving the management of CVT in the Indian context, ultimately enhancing patient care and outcomes. In summary, CVT is a rare but important cause of stroke, particularly among young adults in India. While the mortality rate is lower compared to arterial strokes, the need for targeted research and an improved understanding of CVT in the Indian population remains critical. The present study will contribute valuable insights into the risk factors and outcomes of CVT, supporting the development of effective management strategies and improving the prognosis for affected patients.

## Materials and methods

Study design and setting

This prospective observational research conducted at Marengo Asia Hospital, a tertiary care center in Faridabad, India, spanned from May 2022 to April 2024, covering a period of two years. Data collection was initiated after getting the ethical clearance certificate from the institutional ethical committee of Marengo Asia Hospital (IEC-MAH-2024-001).

Sample Size Calculation

To determine the appropriate sample size for the study with a 95% confidence interval and an 8% margin of error, the following calculation was used. The CVT in the population is estimated at 15% (0.15). The z-value for a 95% confidence level was 1.96. With a margin of error set at 8% (0.08) and the complement of prevalence (Q) being 0.85, the sample size was calculated using the formula: N=Z2∙P∙Q/E2. Thus, the sample size required for the study was approximately 75 patients.

Selection Criteria

Seventy-five individuals diagnosed with CVT were assessed for screening and potential trial inclusion. Participants were eligible for the study if they had CVT, were over 18 years old, and had a diagnosis confirmed through computed tomography (CT)/magnetic resonance imaging (MRI) brain imaging with magnetic resonance venography (MRV). Written informed consent was obtained from all participants. Patients with vascular malformations, infarcts, or conditions resulting from treatments or diagnostic procedures piercing the dura mater were not eligible.

Data sources and variables

Data on the baseline characteristics of the patients and their medical history about CVT risk factors, such as the use of oral contraceptives, were gathered. Following normal recommendations and protocols, all patients received antiepileptic medication, anti-edema measures (mannitol, furosemide, and glycerol), and anticoagulant treatment in the critical care unit. The patients also had MRI brain scans using MRV. Utilizing the Modified Rankin Scale (mRS), the degree of impairment or dependency in day-to-day activities was assessed. Six levels of impairment are described on this scale, with grade 0 representing no symptoms at all and grade 6 representing death. When the mRS score was less than two, it was deemed functionally independent; when it was greater than two, it was deemed functionally dependent. The mRS score was recorded at the time of admission, discharge, and a six-week follow-up.

Laboratory testing, such as coagulation studies, serum homocysteine levels, hemograms, and antinuclear antibody tests, was performed to determine the risk factors for thrombophilia. The World Health Organization (WHO) 2016 criteria were used to diagnose polycythemia vera [7]. The updated Sapporo Classification Criteria, commonly called the Sydney criteria, were used to diagnose antiphospholipid syndrome (APS) [8]. For those under 60, the cut-off value for high blood homocysteine levels was greater than 15 mg/100 ml, while for people over 60, it was greater than 20 mg/100 ml [9].

Statistical analysis

Data analysis and interpretation were conducted using version 21.0 of the IBM SPSS Statistics software for Windows (IBM Corp., Armonk, NY). The analysis involved calculating means and standard deviations and performing Chi-square tests, with a p-value of less than 0.05 deemed statistically significant. The mRS scores at admission and follow-up were compared using the chi-square test with the Glasgow Coma Scale (GCS) at initial assessment, neurological impairments, and intracerebral hemorrhage (ICH).

## Results

Table 1 shows the demographic distribution of the 75 patients included in the study. Of the total, 42 patients (56%) were male and 33 patients (44%) were female. Regarding age, 50 patients (66.7%) were below 40 years, while 25 patients (33.3%) were 40 years or older. The mean age of the patients was 34.5 years with a standard deviation of ±12.3 years.

**Table 1:** The distribution of patients according to demographics

Variables	Frequency (n)	Percentage (%)
Gender		
Male	42	56%
Female	33	44%
Age		
Below 40 years	50	66.7%
40 years and above	25	33.3%
Mean age	34.5 ± 12.3	

Table 2 shows the distribution of etiology among the patients. Polycythemia was the most common etiology, observed in 22 patients (29.3%). Within this group, 11 patients (14.67%) were diagnosed with polycythemia vera, eight patients (10.67%) had a history of cigarette smoking, and three patients (4%) had a history of chronic obstructive pulmonary disease (COPD). Intake of oral contraceptive pills (OCPs) was identified in 14 patients (18.7%), while APS was found in 11 patients (14.7%). Pregnancy and puerperium accounted for seven cases (9.3%), followed by hyperhomocystinaemia in six patients (8%). Infection was identified as the etiology in eight patients (10.7%), malignancies in four patients (5.3%), and paroxysmal nocturnal hemoglobinuria in three patients (4%).

**Table 2:** Distribution of patients according to etiology COPD: chronic obstructive pulmonary disease

Etiology	Frequency (n)	Percentage (%)
Polycythemia	22	29.3%
Diagnosed with polycythemia vera	11	14.67%
Diagnosed with a history of cigarette smoking	8	10.67%
Diagnosed with a history of COPD	3	4%
Intake of oral contraceptive pills	14	18.7%
Antiphospholipid syndrome	11	14.7%
Pregnancy and puerperium	7	9.3%
Hyperhomocystinaemia	6	8%
Infection	8	10.7%
Malignancies	4	5.3%
Paroxysmal nocturnal hemoglobinuria	3	4%

Table 3 shows the distribution of mRS scores among 75 patients at admission and at the six-week follow-up. At admission, 38 patients (50.7%) had an mRS score of ≤ 2, while 37 patients (49.3%) had a score of > 2. At the six-week follow-up, the number of patients with an mRS score of ≤ 2 increased to 54 (72%), and those with a score of > 2 decreased to 21 (28%). The total number of patients remained at 75 at both admission and follow-up, with percentages summing to 100% in each case.

**Table 3:** Patients' mRS scores at initial admission and during the six-week subsequent monitoring mRS: Modified Rankin Scale

mRS Score	At admission (n=75)	Percentage (%)	Follow-up at 6 weeks (n=75)	Percentage (%)
≤2	38	50.7%	54	72%
>2	37	49.3%	21	28%
Total	75	100%	75	100%

Table 4 displays the outcomes associated with GCS scores, neurological impairments, and ICH at the time of admission. Among patients with a mRS score of two or less, 10 patients (13.3%) had a GCS score of less than 15, while 28 patients (37.3%) had a GCS score of 15. In contrast, among patients with an mRS score greater than two, 12 patients (16%) had a GCS score of less than 15, and five patients (6.7%) had a GCS score of 15, with a significant p-value of 0.0001. Regarding neurological deficits, 12 patients (16%) with an mRS score of two or less had neurological impairments, compared to 15 patients (20%) with an mRS score greater than two. The absence of neurological deficits was noted in 26 patients (34.7%) with an mRS score of two or less, while only six patients (8%) with an mRS score greater than two had no neurological impairments, with a p-value of 0.0012. In terms of ICH, 11 patients (14.7%) with an mRS score of two or less had ICH, while 13 patients (17.3%) with an mRS score greater than two had ICH. The absence of ICH was observed in 27 patients (36%) with an mRS score of two or less, compared to eight patients (10.7%) with an mRS score greater than two, with a p-value of 0.0005.

**Table 4:** Outcomes associated with Glasgow Coma Scale (GCS) scores, neurological impairments, and intracerebral hemorrhage at the time of admission mRS: Modified Rankin Scale

Variables	mRS ≤ 2 (n = 75)	Percentage (%)	mRS > 2 (n = 75)	Percentage (%)	Chi-square test	p-value
GCS						
GCS < 15	10	13.3	12	16	28.354	p=0.0001
GCS = 15	28	37.3	5	6.7		
Neurological deficits						
Yes	12	16	15	20	10.540	p=0.0012
No	26	34.7	6	8		
Intracerebral hemorrhage (ICH)						
Yes	11	14.7	13	17.3	15.710	p=0.0005
No	27	36	8	10.7		

Table 5 shows the outcomes for 15 patients who underwent decompressive craniotomy. Of these, 10 patients (13.33%) improved, two patients (2.67%) deteriorated, and one patient (1.33%) expired. Additionally, two patients (2.67%) were lost to follow-up.

**Table 5:** Outcomes of patients who underwent decompressive craniotomy

Outcome	Frequency (n)	Percentage (%)
Total patients	15	20%
Improved	10	13.33%
Deteriorated	2	2.67%
Expired	1	1.33%
Lost to follow-up	2	2.67%

## Discussion

With an average age of 34.5±12.3 years, this study found that a notable proportion of individuals with CVT were in their thirties. This observation is consistent with several studies from both Western and Indian contexts, including those by Ferro et al. [1], Pai et al. [10], Narayan et al. [5], and Wassay et al. [11]. Among the 75 patients, 33 patients (44%) were female and 42 patients (56%) were male, which contrasts with earlier studies that reported a higher percentage of female patients, such as the research by Wassay et al. [11] and Ferro et al. [1]. Historically, gender-specific risk factors, including the use of oral contraceptives, pregnancy, puerperium, and hormone replacement therapy, have been linked to this gender disparity. Nevertheless, the current study's higher proportion of males (42 patients, 56%) corresponds with recent research from India by Pai et al. [10] (male:female ratio of 3:2) and Narayan et al. [5] (male:female ratio of 1.16:1). Changes in the use of less thrombogenic OCPs, such as levonorgestrel and norethisterone [10], or variations in healthcare access for women, may explain the shifting gender trends over the past two decades.

The most prevalent risk factors identified in this study were polycythemia in 22 patients (29.3%) and the use of OCPs in 14 patients (18.7%). Of the 22 patients with polycythemia, 11 patients (50%) were diagnosed with polycythemia vera, eight patients (36.4%) had a history of cigarette smoking, and three patients (13.6%) had a history of COPD. The decreasing incidence of OCPs as a causative factor for CVT in recent years is likely due to the use of fewer thrombogenic OCPs. Antiphospholipid syndrome was identified in 11 patients (14.7%), with most cases being primary APS. Pregnancy and the postpartum period were responsible for seven cases (9.3%) of CVT, with all affected patients experiencing CVT during their third trimester or immediately after childbirth. This can be attributed to the hypercoagulable state associated with pregnancy, which intensifies after delivery. Hyperhomocysteinemia was observed in six patients (8%), while infections were detected in eight patients (10.7%), a rate higher than reported in additional Indian research, including studies by Pai et al. [10] and Narayan et al. [5], possibly due to the hospital's rural location and lack of awareness about these infections.

The most frequent risk factor for CVT, according to the current findings as well as those of Pai et al. [10] and Narayan et al. [5], was polycythemia, whereas OCPs were reported to be the most prevalent by Ferro et al. [1]. Comparable to previous research, pregnant and puerperal individuals had a similar rate of CVT. However, there was a somewhat increased incidence of infections resulting in CVT, maybe as a result of the hospital's remote location. While the risk of cancer in CVT was similar to that of previous Indian research, Wassay and colleagues [11] and Ferro et al. [1] found a greater risk in Western studies, which may have been caused by dietary differences and higher detection rates in industrialized nations. Because vitamin B12 deficiency is more common in the Indian population, the frequency of hyperhomocysteinemia was lower in Western research but similar to other Indian studies. Financial reasons prevented the testing of hereditary thrombophilia markers in the current investigation. Nevertheless, other research, such as that conducted by Ferro et al. [1], Pai and associates [10], Narayan et al. [5], Wassay et al. [11], and others, have demonstrated that hereditary thrombophilia plays a major role in the etiology of CVT.

In the current study, 54 patients (72%) after six weeks of follow-up had attained functional independence (measured by an mRS score of ≤ 2). During the six-week follow-up, 54 patients (72%) had an mRS score of ≤ 2, compared to 38 patients (50.7%) at admission. Of the 15 patients who underwent decompressive craniotomies, 10 patients (13.33%) had improvements, two patients (2.67%) had a decline, and one patient (1.33%) passed away, most likely from cerebral edema and transtentorial herniation. During their hospital stay, 14 patients (18.7%) needed mechanical breathing, and two patients (2.67%) were lost to follow-up. In comparison with other research, Patil et al. [12] found that 84% of individuals were released with either partial or total improvement, 6% required decompressive surgery with similar outcomes, and 16% succumbed to cerebral edema and transtentorial herniation. Thota et al. [13] reported that 20% of patients required decompressive craniectomy, with 70% achieving complete functional recovery and 6% dying during hospitalization. Natarajan et al. [14] reported an 89.6% recovery rate without neurological deficits and a total mortality rate of 6.2%. Ferro et al. [1] noted that 86.6% of individuals returned to a state without neurological impairment deficits, with a mortality rate of 8.3%.

According to the current research, poor outcomes were linked to altered consciousness at presentation, neurological impairments, and intracerebral hemorrhage. Ferro et al. [1] identified comparable prognostic factors, such as age over 37 years, male gender, coma at admission, cognitive impairment, and intracerebral hemorrhage observed on admission CT scans. Likewise, Wassay et al. [11] found that ICH and altered mental status were strong predictors of unfavorable outcomes in CVT cases. Similar results were also reported by Aasish et al. [15], reinforcing the importance of early diagnosis and intervention in improving patient outcomes.

Limitations of the study

The study's limitations include the lack of genetic thrombophilia marker testing due to financial constraints and the potential bias from the hospital's rural setting, which may affect the generalizability of the findings. Further research is needed to explore genetic factors and validate these findings in diverse populations.

## Conclusions

The present findings highlight that CVT predominantly affects younger individuals. Male patients were slightly more affected than females, contrasting with earlier studies that reported a higher incidence in women due to gender-specific risk factors. The primary risk factors identified were polycythemia and the use of oral contraceptive pills, although the latter's incidence is decreasing due to less thrombogenic formulations. Functional independence was achieved in a significant proportion of patients at six-week follow-up, indicating a generally favorable prognosis for CVT with appropriate management. However, altered consciousness, neurological deficits, and ICH at presentation were linked to poorer outcomes.
